# Evaluation of Clinical Efficacy Between Forsus and Advansync Fixed Functional Appliance for the Treatment of Skeletal Class II Malocclusion Using 3D Cone Beam Computed Tomography (CBCT): A Prospective Randomized Clinical Trial

**DOI:** 10.7759/cureus.33399

**Published:** 2023-01-05

**Authors:** Hemanth M, Kiran Shadani, Aravind M, Afshan S W, Suchitra MP, Prajwal P, Shantanu Daksha

**Affiliations:** 1 Orthodontics and Dentofacial Orthopaedics, Dayananda Sagar College of Dental Sciences, Bengaluru, IND

**Keywords:** skeletal class ii malocclusion, cbct, post-pubertal, advansync appliance, forsus appliance

## Abstract

Objectives: The objective is to assess and compare whether AdvanSync gives better skeletal, dental, and soft tissue outcomes than Forsus fixed functional appliance in post-pubertal skeletal Class II malocclusion patients.

Methodology: A prospective study was conducted using 3D-CBCT of patients taken before and after fixed functional appliance therapy. The sample consisted of 16 patients divided into two groups: Group 1 was treated with Forsus and group 2 with AdvanSync appliance. All subjects were in their post-pubertal growth phase. Treatment changes were evaluated between the study groups using 12 angular and 14 linear parameters. The data were subjected to statistical analysis.

Results: Statistically significant changes in SNB (P:0.04) and ANB (P:0.01) in Forsus appliance and AdvanSync (SNB, P:008), (ANB, P: <0.001) were found between the pre and post-fixed functional appliance protocol. The effective mandibular length increased in both groups Forsus(P-value: 0.01) and AdvanSync (P-value: 0.01). Group 1 resulted in lower incisor proclination and intrusion, a reduction in an overbite, whereas group 2 resulted in lower incisor proclination, upper incisor extrusion, and retroclination. Both groups showed significant improvement in the molar relation and overjet. An increase in the total facial convexity was shown in group 1; group 2 showed an increase in the facial convexity and total facial convexity. There was no statistically significant difference between the effects of the Forsus and Advansync appliance groups in the post-fixed functional period. Both groups presented similar results.

Conclusion: Forsus and Advansync appliances are effective with similar results in normalizing skeletal Class II malocclusion in post-pubertal patients. There was no statistically significant difference between the groups using Forsus or AdvanSync. The changes were minimal - mandibular skeletal effects, majorly dentoalveolar effects, and mild soft tissue effects.

## Introduction

The subject of facial aesthetics is pre-eminently important to orthodontists [1]. The current trend of orthodontic diagnosis and treatment planning, treatment objectives, and assessment of treatment outcomes is towards an increasing emphasis on soft tissue relationships rather than underlying hard tissue relations. Evaluation of the patient's soft tissues is now a critical step that begins with the global approach of macro, mini, and microesthetic evaluations; started from the outside in, the evaluation of the soft tissue profile being the first [2].

Class II malocclusion is the most frequently encountered problem as it affects one-third of the population [3]. The most recommended therapeutic approach to Class II malocclusion in post-pubertal patients is using fixed functional appliances [3]. These appliances are classified into rigid, flexible, and hybrid fixed functional appliances. Rigid fixed functional appliances restrict mandibular movement and flexible fixed functional appliances have a frequency of breakage. These shortcomings led to the improvisation of “Hybrid fixed functional appliances” [4]. Of the many fixed functional appliances, Forsus fatigue-resistant devices and AdvanSync are used frequently because of their hybrid nature.

The Forsus Fatigue Resistant Device is one of the various fixed functional devices commonly used [5]. It has more of a dentoalveolar effect on the mandible. One of the major disadvantages is the lower incisor proclination that accompanies the treatment [5].

The AdvanSync2 Class II corrector is another fixed functional appliance. It is modeled on the original Herbst but has a much smaller size, is easier to place, activate, and remove, and most importantly, can be used in conjunction with full arch fixed appliances throughout. There is no need to level and align both arches and use heavy stainless steel stabilizing wires prior to placement of the Class II corrector like in conventional fixed functional appliances [6].

FFAs are the most commonly used appliance to correct the skeletal discrepancy in a growing stage, but still, their enhancement effect on mandibular growth has been questionable. All these FFAs correct the skeletal Class II malocclusion by the combined effects of skeletal and dental changes, including the advancement of the mandible, restricting the growth of the maxilla, fanning the lower anterior, and retroclination of the maxillary anterior [7]. Another literature review on fixed functional appliances suggested that there is a small but statistically and clinically important effect on the skeletal and dentoalveolar parameters of patients treated with fixed functional appliances [8].

Traditionally, lateral and frontal cephalometric radiographs have been used to determine craniofacial discrepancies and deformities, with the analysis based on a series of cephalometric points. Problems associated with conventional cephalograms, e.g., errors in patient position, differential magnification of bilateral structures, and superimposition of craniofacial structures complicate the precise localization of cephalometric landmarks. CBCT is an alternative method for the assessment of craniofacial relationships of orthodontic and surgical patients overcoming the problem of superimposition and magnification errors seen in conventional cephalograms [9].

Forsus and Advansync fixed functional appliances have been studied individually [5,6], but there is a lack of studies comparing the effects of both. This study aims to compare whether Advansync gives better skeletal, dental, and soft tissue outcomes than Forsus fixed functional appliance for the treatment of skeletal class II malocclusion in post-pubertal patients using 3D CBCT.

## Materials and methods

This prospective clinical study was conducted at the Department of Orthodontics and Dentofacial Orthopedics, Dayananda Sagar College of Dental Sciences, Bangalore, India. The study was approved by Institutional Review Board and Ethical approval was obtained.

Materials

Forsus fatigue resistant device (3M Unitek), Advansync fixed functional appliance (Ormco), 3D CBCT - J Morita 3D Accutome (FoV: 17*10), fixed mechanotherapy using MBT brackets .022 slot, stainless steel (SS), Nitinol (NiTi) wires, NemoCeph 3D software.

Inclusion criteria

(1) Post-pubertal patients between the age group of 11 and 16 years. (2) Skeletal class II subjects with Retrognathic mandible. (3) Overjet of at least 5 mm. (4) Positive visual treatment objective (VTO).

Exclusion criteria

(1) Patients with no scope for further growth. (2) History of orthodontic treatment. (3) Severe proclination and crowding of anterior teeth. (4) Any systemic disease affecting bone and general health. Oral and written explanations of the purpose of the study were given to all subjects who agreed to participate. Informed consent was taken from all the participants.

Interventions

**Figure 1:**
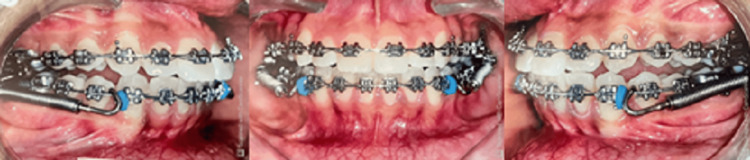
Group 1 with Forsus appliance

**Figure 2:**
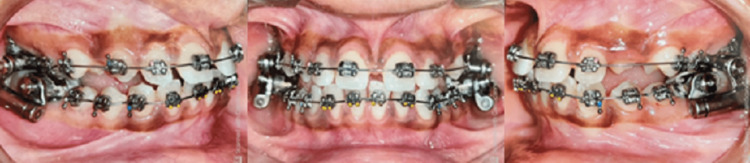
Group 2 with AdvanSync appliance

The study included a total of 16 subjects, in their post-adolescent age ranging between 11 and 16 years. The subjects were divided randomly into two groups of 8 each based on the computer-generated sequence: Group 1 consisted of patients treated with Forsus Fatigue Resistant Device (Figure 1) and Group 2 (Figure 2) consisted of patients treated with Advansync appliance.

**Figure 3:**
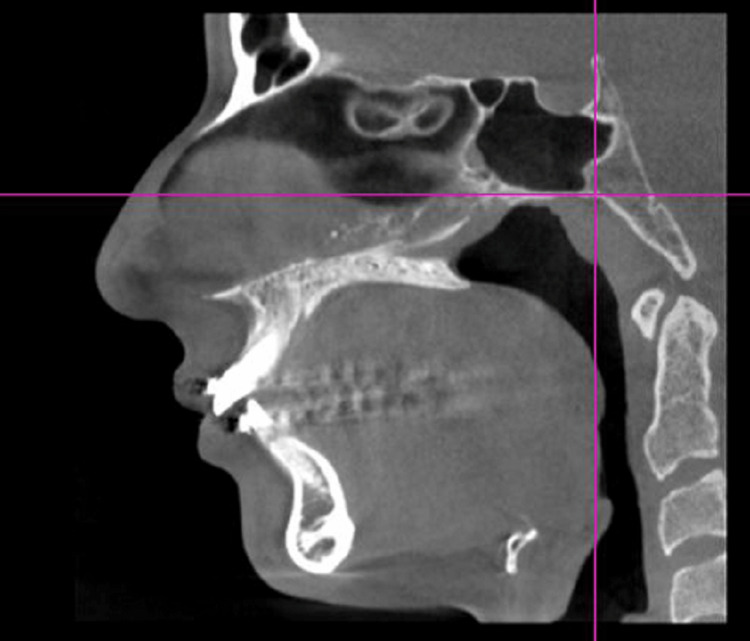
CBCT pre-treatment with Forsus CBCT - Cone beam computed tomography

**Figure 4:**
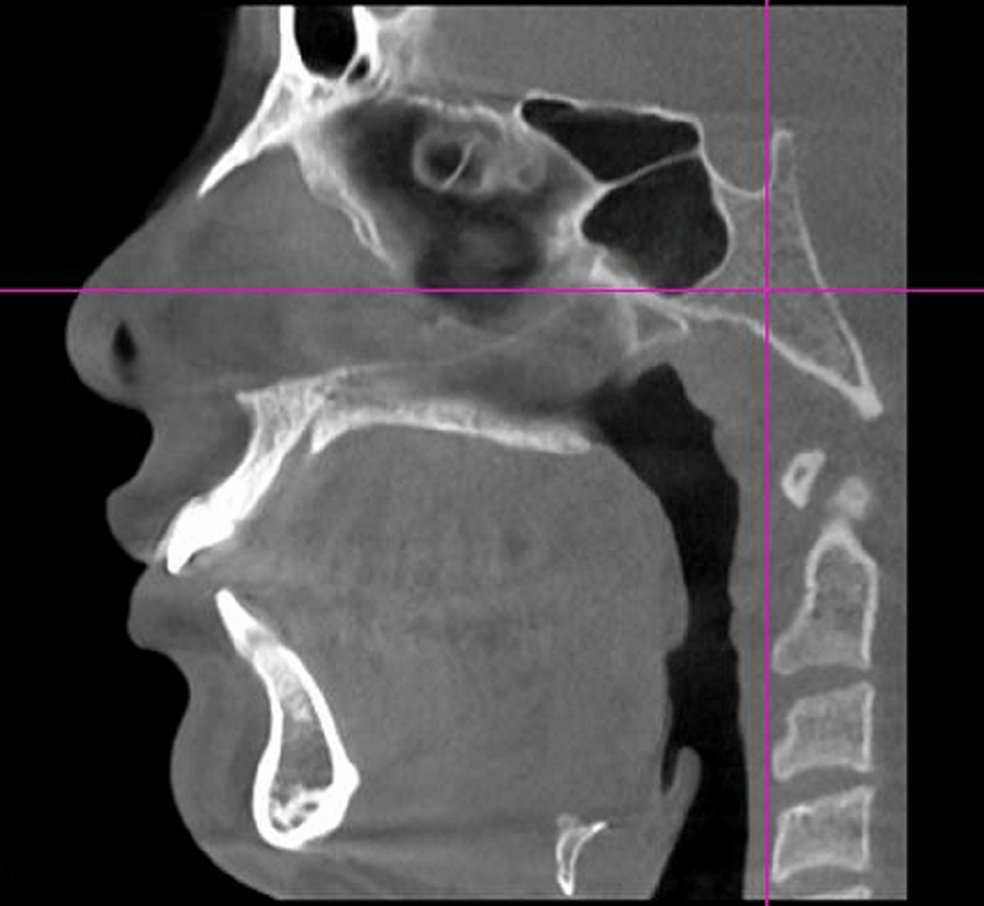
CBCT pretreatment with AdvanSync CBCT - Cone beam computed tomography

CBCT scans of 16 subjects were taken before the placement of the appliance and after the fixed functional appliance therapy. The data was recorded using J Morita 3D Accutome (FoV: 17*10, 5mA, 90 kV, slice thickness:1mm, slice interval:0.5 mm) at two time periods, one before placement of the fixed functional appliance (T1) and post-fixed functional appliance treatment (T2) (Figures 3, 4).

Forsus Appliance Group

The treatment was started by bonding the upper and lower arches with 0.022*0.028 MBT prescription (Figure 1). Leveling and alignment of the arches were achieved using initial round NiTi wires, rectangular NiTi archwire followed by rectangular stainless steel archwire. Once 19*25 SS was engaged, prefixed functional appliance CBCT was taken.

**Figure 5:**
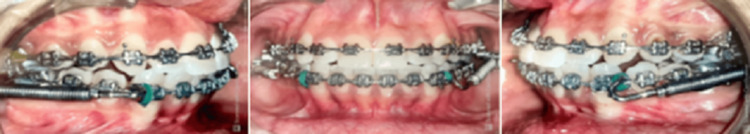
Post-treatment with Forsus appliance

**Figure 6:**
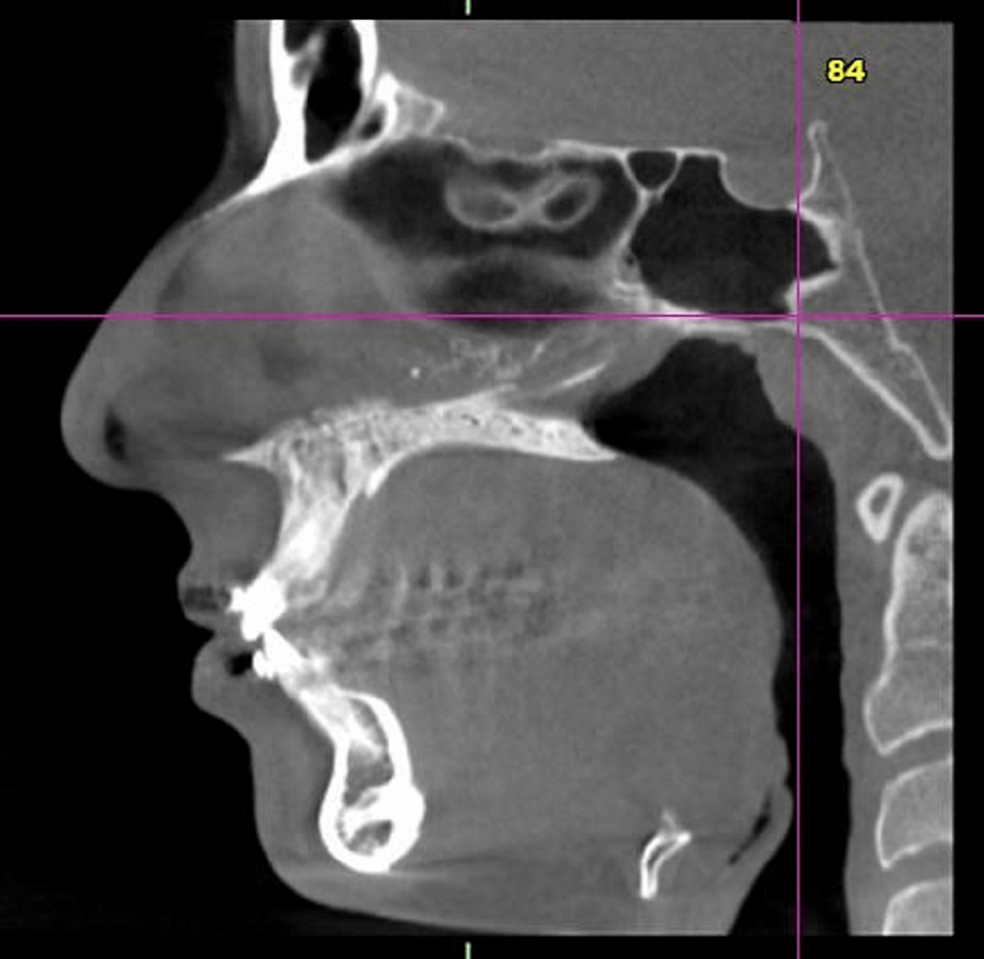
Post-Forsus treatment CBCT CBCT - Cone Beam Computed Tomography

Forsus appliance was delivered with 0.019* 0.025” SS as the working archwire. The appropriate size of the appliance was selected for each patient using the measurement gauge supplied by 3M Unitek. The upper assembly was engaged in the headgear tube of the upper molar band and the push rod was engaged to the lower main archwire distal to the canine. Once the desired corrections were achieved, a post-fixed functional appliance CBCT was taken (Figures 5, 6).

AdvanSync Appliance Group

**Figure 7:**
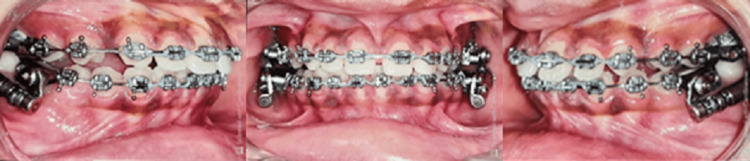
Post-treatment with AdvanSync appliance

**Figure 8:**
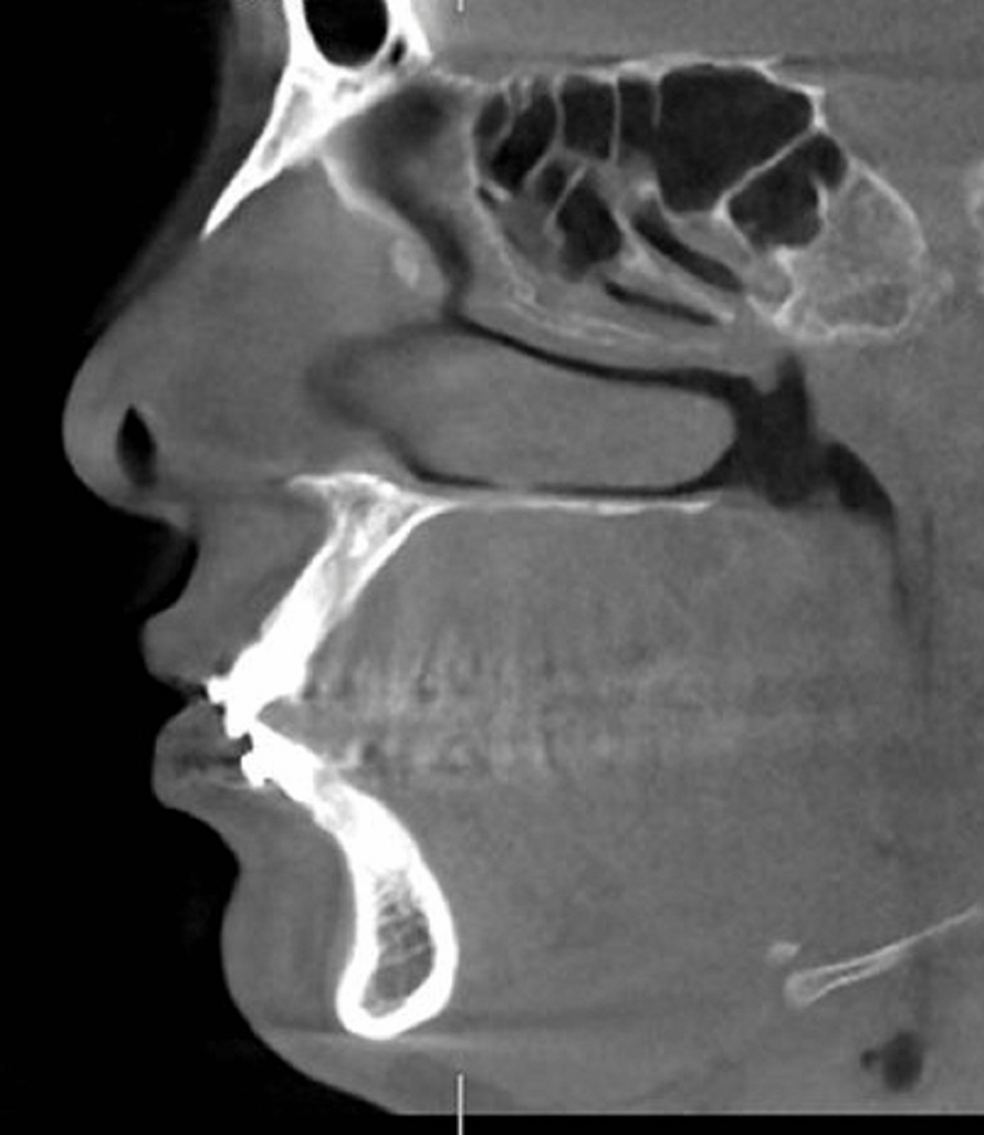
Post treatment with AdvanSync CBCT CBCT - Cone Beam Computed Tomography

CBCT was taken at the beginning of the treatment, before placement of the appliance (Figure 3). Separators were placed for the upper and lower molars and the appropriate size of the crown was selected for the individual patients. The crowns were cemented to the molars using glass ionomer cement followed by the engagement of the telescopic arms to the crown assembly using the driver supplied by the manufacturer; with simultaneous bonding of upper and lower teeth with 0.022* 0.028 MBT prescription from the second premolar to second premolar teeth. The post-fixed functional appliance CBCT was taken when desired corrections were achieved after 7-8 months (Figures 7, 8).

After taking the scans, the scans were converted into digital imaging and communication in medicine (DICOM) format. The data were reconstructed into 3D images using Nemoceph 3D software. The anatomical landmarks were identified on 3D CBCT multiplanar and reconstructed images. The 3D images were oriented based on the midsagittal reference line formed by soft tissue nasion, subnasale and soft tissue pogonion. A lateral image was extracted, and various linear and angular measurements of skeletal, dental, and soft tissues were performed for both groups. The cephalometric linear and angular measurements assessed were as follows.

Skeletal Angular

SNA (angle drawn between sella-nasion to point A), SNB (angle drawn between sella-nasion to point B), ANB (angle drawn between point A to nasion and point B to nasion), GoGn- SN Plane (angle drawn between Gonion-Gnathion plane to Sella-Nasion plane), MPA (Mandibular plane angle), Gonial angle (the angle formed by the base of the mandible and posterior border of ramus), Beta angle (the angle formed between the A-B line and the perpendicular through point A from the apparent axis of the condyle).

Skeletal Linear

A perp N (line drawn from point A perpendicular to Nasion), ANS perp N (line drawn from anterior nasal spine perpendicular to nasion), ANS TO PNS (line drawn from anterior nasal spine to posterior nasal spine); Dental angular: U1-SN (upper incisor to sell-nasion), L1-SN (lower incisor to sell-nasion), L1-MP (lower incisor to the mandibular plane); Dental linear: U6-NF (line drawn between upper 1st molar to the nasal floor), L6-MP (line drawn between lower 1st molar to the mandibular plane); Molar Correction, U6-NF (angle drawn from upper molar to nasal floor), L1-MP (angle drawn from lower incisor to mandibular plane), Overjet, Overbite and; Soft tissue angular: Angle Of Total Facial Convexity, Facial Convexity, Nasolabial angle, and Mentolabial Angle.

Sample size calculation

Based on the mean and SD of group 1 and group 2 based on the studies of Arora et al. [10], the effect size was calculated and found to be 1.34, utilizing this effect size as input value in the G power software, the minimum sample size calculated was 16, with eight subjects in each group. The input data are as follows

1. A priori: Compute the required sample size 2. Tail(s) = One 3. Effect size d = 1.34 4. α err prob = 0.05 5. Power (1-β err prob) = 0.80 6. Allocation ratio N2/N1 = 1 7. Output: The minimum sample size required for this study was 16 and each group contained eight subjects. 

Statistical analysis

Descriptive and analytic statistics were obtained using Statistical Package for the Social Sciences (SPSS) software, version 22.0 (IBM Corp., Armonk, NY). The significance level was P < 0.05. The descriptive analysis includes expression of Skeletal, Dental and Facial Soft Tissue Parameters in terms of Mean and SD for each study group. 

Independent Student t-Test and Mann Whitney U-Test were used to compare the Mean Skeletal, Dental and Facial Soft Tissue Parameters between two groups at Pre and Post Treatment Periods. Student Paired t-Test and Wilcoxon Signed Rank Test was used to compare the Mean Skeletal, Dental and Facial Soft Tissue Parameters between Pre and Post-Treatment Period in each study group.

## Results

This study was done to assess the clinical efficacy of Forsus and Advansync fixed functional appliance for the treatment of skeletal Class II malocclusion patients in their post-pubertal growth phase using three-dimensional CBCT. The objectives were: (1) To assess the skeletal, dental and soft tissue changes brought about by Forsus fixed functional device, (2) To assess the skeletal, dental and soft tissue changes brought about by AdvanSync fixed functional device. 3) To compare the skeletal, dental and soft tissue changes of AdvanSync and Forsus fixed functional appliance at the end of functional treatment Participant Flow: A total of 16 patients were recruited in the study.

Pretreatment comparison between Forsus and AdvanSync groups

Baseline Data

Two groups were matched for the pre-treatment skeletal and dental malocclusion, severity of the case and the soft tissue parameters. Table 1 represents the comparison of mean values of Angular Skeletal Parameters between the two groups at Pretreatment and suggests that there was no statistically significant difference in the SNA (P:0.17), SNB (P:0.38), ANB (P:0.89), MPA (P:0.70) and Gonial angle (P:0.41) at the baseline, when the level of significance was set at P<0.05).

**Table 1:** Comparison of mean values of angular skeletal parameters between two groups at pre Rx period using independent student t-test SD: Standard deviation, SNA: Sella -nasion -point a, SNB: Sella -nasion- point B, ANB: point a- nasion- point B, MPA: mandibular plane angle

Parameters	Group	N	Mean	SD	Mean Diff	P-value
SNA	Group 1	8	83.659	2.467	1.435	0.17
	Group 2	8	82.224	1.371		
SNB	Group 1	8	78.469	2.813	1.088	0.38
	Group 2	8	77.381	1.951		
ANB	Group 1	8	4.940	1.193	0.098	0.89
	Group 2	8	4.843	1.480		
MPA	Group 1	8	24.685	5.726	0.981	0.70
	Group 2	8	23.704	4.133		
Gonial Angle	Group 1	8	119.221	7.346	-2.450	0.41
	Group 2	8	121.671	3.344		

Table 2 represent the comparison of mean values of Linear Skeletal Parameters between the two groups at Pretreatment and suggest that there were no statistically significant difference in the N perp Point A (P:0.71), N perp ANS (P:0.96), ANS-PNS (P:0.08), N perp Point B (P:0.88), N perp Pog (P:0.96), Co-Gn (P:0.23) and Go-Gn (P:0.71) values, when the level of significance was set at P<0.05.

**Table 2:** Comparison of mean values of linear skeletal parameters between two groups at pre Rx period using independent student t-test A PERP N: Point A perpendicular to Nasion, ANS PERP N:  Anterior Nasal Spine perpendicular to Nasion, ANS-PNS: Anterior Nasal Spine-Posterior Nasal Spine, POG-PERP-N: Pogonion -perpendicular-Nasion, B-perp: B Perpendicular, CO-GN: Condylion- Gnathion, GO-GN: Gonion- Gnathion

Parameters	Group	N	Mean	SD	Mean Diff	P-value
A PERP N	Group 1	8	-0.425	3.127	-0.596	0.71
	Group 2	8	0.171	4.770		
ANS PERP N	Group 1	8	4.593	1.638	-0.148	0.96
	Group 2	8	4.740	2.720		
ANS-PNS	Group 1	8	53.650	3.441	3.550	0.08
	Group 2	8	50.100	3.863		
POG PERP N	Group 1	8	-4.865	6.835	-0.799	0.96
	Group 2	8	-4.066	6.951		
B PERP	Group 1	8	-5.393	7.490	-0.638	0.88
	Group 2	8	-4.755	5.917		
CO-GN	Group 1	8	104.896	6.880	3.779	0.23
	Group 2	8	101.118	2.562		
GO-GN	Group 1	8	68.984	7.447	1.840	0.71
	Group 2	8	67.144	3.644		

Table 3 shows that there was no statistically significant difference between the two groups when comparing the pretreatment Angular dental parameters: U1-SN (P:0.37), L1-SN (P:0.52) and L1-MP (P:0.56) values. Table 3 also shows that there were no statistically significant difference between the two groups when comparing the pretreatment Linear dental parameters in vertical plane which included U1-NF (P:0.07), U6-NF (P:0.66), L1-MP (P:0.07), L6- MP (P:0.41), and in sagittal plane which included Molar correction (P:0.35), Overjet (P:0.18) and Overbite (P:0.26), when the level of significance was set at P<0.05.

**Table 3:** Comparison of mean values of angular and linear dental parameters between two groups at Pre Rx Period using Independent Student t-Test U1 -SN: upper incisor to sella-nasion, L1 - SN: lower incisor to sella- nasion, L1-MP: lower incisor- mandibular plane, U1-NF: upper incisor- nasal floor, U6-NF: upper molar- nasal floor, L6-MP: lower molar- mandibular plane

Parameters	Group	N	Mean	SD	Mean Diff	P-value
U1-SN	Group 1	8	109.860	7.487	-3.578	0.37
	Group 2	8	113.438	7.909		
L1-SN	Group 1	8	48.356	9.054	-2.683	0.52
	Group 2	8	51.039	7.109		
L1-MP	Group 1	8	99.766	4.128	-1.945	0.56
	Group 2	8	101.711	8.236		
U1-NF	Group 1	8	25.873	2.071	2.073	0.07
	Group 2	8	23.800	2.088		
U6-NF	Group 1	8	21.110	3.233	0.696	0.66
	Group 2	8	20.414	2.967		
L1-MP	Group 1	8	39.914	3.403	3.235	0.07
	Group 2	8	36.679	2.745		
L6-MP	Group 1	8	30.065	3.116	1.686	0.41
	Group 2	8	28.379	4.634		
Molar correction	Group 1	8	2.295	1.280	0.490	0.35
	Group 2	8	1.805	0.672		
Overjet	Group 1	8	5.940	0.728	-1.388	0.18
	Group 2	8	7.328	2.709		
Overbite	Group 1	8	4.166	0.829	1.228	0.26
	Group 2	8	2.939	2.852		

Table 4 represents the mean values of Angular Facial Soft Tissue Parameters between two groups at Pretreatment and suggest that there was no significant difference in the facial convexity angle (P:0.65), total facial convexity angle (P:0.41), Nasolabial (P:0.16) and Mentolabial angle (P:0.32) at the baseline. The results suggest that there was no significant difference between the type of cases that were randomly allocated in the two groups. The pretreatment skeletal, dental and soft tissue baseline data was similar for both the treatment groups thereby reducing the selection bias in the study. 

**Table 4:** Comparison of mean values of angular facial soft tissue parameters between two groups at Pre Rx Period using Independent Student t-Test

Parameters	Group	N	Mean	SD	Mean Diff	P-value
Facial convexity	Group 1	8	156.098	4.148	-1.134	0.65
	Group 2	8	157.231	5.395		
Total facial convexity	Group 1	8	126.564	6.008	-2.431	0.41
	Group 2	8	128.995	5.368		
Nasolabial angle	Group 1	8	107.503	9.400	6.860	0.16
	Group 2	8	100.643	9.247		
Mentolabial sulcus	Group 1	8	114.076	16.148	10.577	0.32
	Group 2	8	103.499	23.719		

Post-treatment intergroup comparison between Forsus group and AdvanSync group

The comparison of mean values of Angular Skeletal Parameters at Post-treatment suggests that there was no statistically significant difference in the SNA (P:0.25), SNB (P:0.61), ANB (P:0.63), MPA (P:0.97) and Gonial angle (P:0.36) when the treatment results between Forsus group and AdvanSync appliance group were compared (Table 5). This shows that both groups had similar results with the above angular parameters.

**Table 5:** Comparison of mean values of Angular Skeletal Parameters between 2 groups at Post Rx Period using Independent Student t-Test SNA : Sella -nasion -point a, SNB : Sella -nasion- point B, ANB : point a- nasion- point B, MPA : mandibular plane angle

Parameters	Group	N	Mean	SD	Mean Diff	P-value
SNA	Group 1	8	83.089	2.209	1.175	0.25
	Group 2	8	81.914	1.633		
SNB	Group 1	8	79.173	3.253	0.749	0.61
	Group 2	8	78.424	2.401		
ANB	Group 1	8	3.916	1.866	0.430	0.63
	Group 2	8	3.486	1.612		
MPA	Group 1	8	24.479	4.798	-0.081	0.97
	Group 2	8	24.560	4.328		
Gonial Angle	Group 1	8	120.030	7.041	-2.961	0.36
	Group 2	8	122.991	5.302		

Table 6 represents the comparison of mean values of Linear Skeletal Parameters at Post treatment between Forsus and Advansync group: The results suggests that there was no statistically significant difference in the Point A perp N (P:0.43), ANS perp N (P:0.10), ANS-PNS (P:0.10), Pog perp N (P:0.88), Point B perp (P:0.96), Co-Gn (P:0.29) and Go-Gn (P:0.75) values when the treatment changes between the groups were evaluated. The results showed that both the forsus group and advansync group produce similar linear skeletal parameters.

**Table 6:** Comparison of mean values of linear skeletal parameters between two groups at Post Rx Period using Mann-Whitney U Test A PERP N: Point A perpendicular to Nasion, ANS PERP N:  Anterior Nasal Spine perpendicular to Nasion, ANS-PNS: Anterior Nasal Spine-Posterior Nasal Spine, POG-PERP-N: Pogonion -perpendicular-Nasion, B-perp: B Perpendicular, CO-GN: Condylion- Gnathion, GO-GN: Gonion- Gnathion

Parameters	Group	N	Mean	SD	Mean Diff	P-value
A PERP N	Group 1	8	0.536	3.621	1.071	0.43
	Group 2	8	-0.535	5.005		
ANS PERP N	Group 1	8	5.560	2.573	1.739	0.10
	Group 2	8	3.821	2.710		
ANS-PNS	Group 1	8	53.451	3.532	3.060	0.10
	Group 2	8	50.391	3.859		
POG PERP N	Group 1	8	-2.401	9.412	0.983	0.88
	Group 2	8	-3.384	5.225		
B PERP	Group 1	8	-3.963	8.038	1.163	0.96
	Group 2	8	-5.125	5.143		
CO-GN	Group 1	8	106.020	7.498	3.444	0.29
	Group 2	8	102.576	2.579		
GO-GN	Group 1	8	69.253	7.399	2.189	0.75
	Group 2	8	67.064	3.507		

The comparison of mean values of Angular Dental Parameters between two groups resulted in no significant difference in the U1-SN (P:0.88), L1-SN (P:0.63) and L1-MP (P:0.23) values. The mean values of Linear dental parameters between the two groups showed no statistically significant differences in the parameters in the vertical plane U1-NF (P:0.82), U6-NF (P:0.46), L1-MP (P:0.61), L6-MP (P:0.09), Molar correction (P:0.26), Overjet (P:0.45), Overbite (P:0.16) (Table 7). The result showed that both Forsus and AdvanSync groups produced similar results related to angular dental parameters.

**Table 7:** Comparison of mean values of angular and linear dental parameters between two groups at Post Rx Period using Independent Student t-Test U1-SN: upper incisor to sella-nasion, L1-SN: lower incisor to sella- nasion, L1-MP: lower incisor- mandibular plane, U1-NF: upper incisor- nasal floor, U6-NF:upper molar- nasal floor, L6-MP: lower molar- mandibular plane

Parameters	Group	N	Mean	SD	Mean Diff	P-value
U1-SN	Group 1	8	107.949	8.979	-0.694	0.88
	Group 2	8	108.643	8.304		
L1-SN	Group 1	8	44.923	10.292	-2.356	0.63
	Group 2	8	47.279	8.823		
L1-MP	Group 1	8	104.055	4.394	-4.268	0.23
	Group 2	8	108.323	8.530		
U1-NF	Group 1	8	24.399	5.802	-0.520	0.82
	Group 2	8	24.919	2.459		
U6-NF	Group 1	8	21.689	2.939	0.986	0.46
	Group 2	8	20.703	2.228		
L1-MP	Group 1	8	38.491	3.161	0.716	0.61
	Group 2	8	37.775	2.216		
L6-MP	Group 1	8	30.168	2.667	2.346	0.09
	Group 2	8	27.821	2.501		
Molar correction	Group 1	8	-1.858	0.951	0.616	0.26
	Group 2	8	-2.474	1.122		
Overjet	Group 1	8	2.821	0.902	-0.461	0.45
	Group 2	8	3.283	1.433		
Overbite	Group 1	8	2.350	0.449	-0.629	0.16
	Group 2	8	2.979	1.097		

Table 8 represents the mean values of Angular Facial Soft Tissue Parameters between Forsus groups and Advansync groups at Post treatment and suggest that there was no significant difference in the Facial convexity angle (P:0.42), Total facial convexity angle (P:030), Nasolabial (P:0.12) and Mentolabial angle (P:0.14) at the end of fixed functional appliance therapy. The results showed that both Forsus and AdvanSync appliance produced similar angular facial soft tissue parameters. 

**Table 8:** Comparison of mean values of angular facial soft tissue parameters between two groups at post Rx period using independent student t-test

Parameters	Group	N	Mean	SD	Mean Diff	P-value
Facial convexity	Group 1	8	157.414	5.263	-2.220	0.42
	Group 2	8	159.634	5.480		
Total facial convexity	Group 1	8	128.441	6.112	-3.106	0.30
	Group 2	8	131.548	5.298		
Nasolabial angle	Group 1	8	107.898	7.320	8.034	0.12
	Group 2	8	99.864	11.556		
Mentolabial sulcus	Group 1	8	117.393	12.185	13.303	0.14
	Group 2	8	104.090	20.313		

The results of our study showed that there was no significant difference between the two groups at post treatment. Both Forsus appliance and AdvanSync appliance produce similar skeletal, dental and soft tissue treatment outcomes.

Intragroup pre and post-treatment comparison: Forsus group (group 1)

Table 9 represents the comparison of mean values of Angular Skeletal Parameters between Pre & Post treatment in Forsus group (Group 1). The SNB angle increased from 78.46 ± 2.81 to 79.17 ± 3.25 degrees, which was statistically significant (P:0.04). Also, the ANB angle reduced from 4.94 ± 1.19 to 3.91 ± 1.86 degrees, which was found to be statistically significant (P:0.01). The pretreatment and post treatment SNA angle was 83.65 ± 2.46 and 83.08 ± 2.20, respectively, with no statistical significance. (P:0.09). The MPA was measured to be 24.68 ± 5.72 and 24.47 ± 4.79 at pretreatment and post treatment, respectively. This difference was not statistically significant (P:0.76). The Gonial angle changed from 119.22 ± 7.34 to 120.03 ± 7.04 degrees, with no statistical significance (P:0.44). The results suggest that there was a significant improvement in the skeletal parameters - SNB and ANB angle in the Forsus group.

**Table 9:** Comparison of mean values of angular skeletal parameters between pre Rx & post Rx period in Group 1 using student paired t-test SNA : Sella -nasion -point a, SNB : Sella -nasion- point B, ANB : point a- nasion- point B, MPA : mandibular plane angle

Parameters	Time	N	Mean	SD	Mean Diff	P-value
SNA	Pre Rx	8	83.659	2.467	0.570	0.09
	Post Rx	8	83.089	2.209		
SNB	Pre Rx	8	78.469	2.813	-0.704	0.04*
	Post Rx	8	79.173	3.253		
ANB	Pre Rx	8	4.940	1.193	1.024	0.01*
	Post Rx	8	3.916	1.866		
MPA	Pre Rx	8	24.685	5.726	0.206	0.76
	Post Rx	8	24.479	4.798		
Gonial Angle	Pre Rx	8	119.221	7.346	-0.809	0.44
	Post Rx	8	120.030	7.041		

Table 10 shows the mean values of Linear Skeletal Parameters between Pre Rx & Post Rx in Group 1. Based on the derived values with descriptive statistics, the mean values, standard deviations for Linear dental parameters, Point A perp to N value changed from -0.42 ± 3.12 to 0.53 ± 3.62 mm, with no statistical significance (P:0.24). The ANS perp. N value increased from 4.593 ± 1.63 mm to 5.56 ± 2.57 mm, which was statistically significant (P value: 0.04). On comparing the difference between pre and post Rx value of ANS-PNS, the value changed from 53.65 ± 3.44 to 53.45 ± 3.53 mm respectively, with no statistical significance (0.33). The Pog perp. N value increased from -4.86 ± 6.83 mm to -2.40 ± 9.41 mm, which was not statistically significant (P:0.12). The change in the Point B perp. N from -5.39 ± 7.49 mm to -3.96 ± 8.03 mm, with no statistical significance (P:0.06). The effective condylar length Co-Gn increased from 104.89 ± 6.88 mm to 106.02 ± 7.49 mm, which was statistically significant (P value: 0.01). On comparing the difference between pre and post Rx value of Go-Gn, the value changed from 68.98 ± 7.44 to 69.25 ± 7.39 mm respectively, with no statistical significance (P:0.58).

The results suggest that there was a significant change in ANS perp N value which is statistically significant but not clinically significant. There was significant increase in the effective condylar length in the post appliance phase (CO-GN, P:0.01).

**Table 10:** Comparison of mean values of linear skeletal parameters between pre Rx & post Rx period in Group 1 using Wilcoxon signed rank test A PERP N: Point A perpendicular to Nasion, ANS PERP N:  Anterior Nasal Spine perpendicular to Nasion, ANS-PNS: Anterior Nasal Spine-Posterior Nasal Spine, POG-PERP-N: Pogonion -perpendicular-Nasion, B-perp: B Perpendicular, CO-GN: Condylion- Gnathion, GO-GN: Gonion- Gnathion

Parameters	Time	N	Mean	SD	Mean Diff	P-value
A PERP N	Pre Rx	8	-0.425	3.127	-0.961	0.24
	Post Rx	8	0.536	3.621		
ANS PERP N	Pre Rx	8	4.593	1.638	-0.967	0.04*
	Post Rx	8	5.560	2.573		
ANS-PNS	Pre Rx	8	53.650	3.441	0.199	0.33
	Post Rx	8	53.451	3.532		
POG PERP N	Pre Rx	8	-4.865	6.835	-2.464	0.12
	Post Rx	8	-2.401	9.412		
B PERP	Pre Rx	8	-5.393	7.490	-1.430	0.06
	Post Rx	8	-3.963	8.038		
CO-GN	Pre Rx	8	104.896	6.880	-1.124	0.01*
	Post Rx	8	106.020	7.498		
GO-GN	Pre Rx	8	68.984	7.447	-0.269	0.58
	Post Rx	8	69.253	7.399		

Dental Angular Parameter

U1-SN angle reduced from 109.86 ± 7.48 to 107.94 ± 8.97 degrees, with no statistical significance (P:0.23). L1-SN angle reduced from 48.35 ± 9.05 to 44.92 ± 10.29 degrees, but the changes were not statistically significant (P:0.10). The pre and post L1-MP value increased from 99.76 ± 4.12 to 104.05 ± 4.39 degrees respectively, which was statistically significant (P: <0.001). The results suggest that there was significant amount of lower incisor proclination observed post Forsus appliance therapy.

Dental Linear Parameter

U1-NF value changed from 25.87 ± 2.07 to 24.39 ± 5.80 mm. with no statistical significance (P:0.47). The U6-NF value increased from 21.11 ± 3.23 to 21.68 ± 2.93 mm which was not statistically significant (P:0.190. The L1-MP value reduced from 39.91 ± 3.40 to 38.49 ± 3.16 mm and this difference was found to be statistically significant (P:0.04). L6-MP value changed from 30.06 ± 3.11 to 30.16 ± 2.66 mm, with no statistical significance (P:0.87). The value for Molar correction changed from 2.29 ± 1.28 to -1.85 ± 0.95 mm, which showed highly significant difference (P: <0.001). The change in the Overjet was found to be highly significant which reduced from 5.94 ± 0.72 to 2.82 ± 0.90 mm (P: <0.001). Similarly, the Overbite changed from 4.16 ± 0.89 to 2.35 ± 0.44 mm which is statistically significant (P: <0.001).

Comparing pre and post treatment effects, Forsus appliance resulted in significant intrusion of lower incisors, improvement in the molar relationship, decrease in the overjet and decrease in the overbite. Table 11 shows comparison of mean values of Angular and linear Dental Parameters between Pre Rx & Post Rx in Group 1.

**Table 11:** Comparison of mean values of angular and linear dental parameters between pre Rx & post Rx period in Group 1 using student paired t-test U1 -SN: upper incisor to sella-nasion, L1 - SN: lower incisor to sella- nasion, L1-MP: lower incisor- mandibular plane, U1-NF: upper incisor- nasal floor, U6-NF: upper molar- nasal floor, L6-MP: lower molar- mandibular plane

Parameters	Time	N	Mean	SD	Mean Diff	P-value
U1-SN	Pre Rx	8	109.860	7.487	1.911	0.23
	Post Rx	8	107.949	8.979		
L1-SN	Pre Rx	8	48.356	9.054	3.434	0.10
	Post Rx	8	44.923	10.292		
L1-MP	Pre Rx	8	99.766	4.128	-4.289	<0.001*
	Post Rx	8	104.055	4.394		
U1-NF	Pre Rx	8	25.873	2.071	1.474	0.47
	Post Rx	8	24.399	5.802		
U6-NF	Pre Rx	8	21.110	3.233	-0.579	0.19
	Post Rx	8	21.689	2.939		
L1-MP	Pre Rx	8	39.914	3.403	1.423	0.04*
	Post Rx	8	38.491	3.161		
L6-MP	Pre Rx	8	30.065	3.116	-0.102	0.87
	Post Rx	8	30.168	2.667		
Molar correction	Pre Rx	8	2.295	1.280	4.153	<0.001*
	Post Rx	8	-1.858	0.951		
Overjet	Pre Rx	8	5.940	0.728	3.119	<0.001*
	Post Rx	8	2.821	0.902		
Overbite	Pre Rx	8	4.166	0.829	1.816	<0.001*
	Post Rx	8	2.350	0.449		

Soft Tissue Angular Measurements

The facial convexity angle changed from 156.09 ± 4.14 to 157.41 ± 5.26 degrees, which did not show statistically significant change (P:0.13). The total facial convexity increased from 126.56 ± 6.00 to 128.44 ± 6.11 degrees, which was statistically significant (P:0.001). The Nasolabial angle changed from 107.50 ± 9.40 to 107.89 ± 7.32 degrees, with no statistical significance (P:0.77). The Mentolabial angle changed from 114.07 ± 16.14 to 117.39 ± 12.18 degrees which had no statistical significance (P:0.26).

The results suggest that Forsus appliance brought about significant change in the total facial convexity angle, whereas no change was found in the Facial convexity, Nasolabial and Mentolabial angle (Table 12).

**Table 12:** Comparison of mean values of angular facial soft tissue parameters between pre Rx & post Rx period in Group 1 using student paired t-test

Parameters	Time	N	Mean	SD	Mean Diff	P-value
Facial convexity	Pre Rx	8	156.098	4.148	-1.316	0.13
	Post Rx	8	157.414	5.263		
Total facial convexity	Pre Rx	8	126.564	6.008	-1.878	0.001*
	Post Rx	8	128.441	6.112		
Nasolabial angle	Pre Rx	8	107.503	9.400	-0.395	0.77
	Post Rx	8	107.898	7.320		
Mentolabial sulcus	Pre Rx	8	114.076	16.148	-3.316	0.26
	Post Rx	8	117.393	12.185		

Intragroup pre and post-treatment comparison: AdvanSync appliance

Skeletal Angular Parameter

Table 13 represent the comparison of mean values of Angular Skeletal Parameters between Pre and Post-treatment in Group 2. The pretreatment and post treatment SNA angle was 82.22 ± 1.37 and 81.91 ± 1.63, respectively, with no statistical significance (P:0.19). The SNB angle increased from 77.38 ± 1.95 to 78.42 ± 2.40 degrees, which was statistically significant (P:008). Also, the ANB angle reduced from 4.83 ± 1.48 to 3.48 ± 1.61 degrees, which was highly significant (P: <0.001). The MPA was measured to be 23.70 ± 4.13 and 24.56 ± 4.32 degrees at pretreatment and post treatment, respectively. This difference was not statistically significant (P:0.11). The Gonial angle changed from 121.67 ± 3.34 to 122.99 ± 5.30 degrees, with no statistical significance (P:0.13). The results suggest that similar to Forsus appliance, AdvanSync appliance resulted in significant change in the SNB and ANB angle.

**Table 13:** Comparison of mean values of angular skeletal parameters between pre Rx & post Rx period in Group 2 using student paired t-test SNA: Sella -nasion -point a, snb:sella -nasion- point B, ANB : point a- nasion- point B, MPA : mandibular plane angle

Parameters	Time	N	Mean	SD	Mean Diff	P-value
SNA	Pre Rx	8	82.224	1.371	0.310	0.19
	Post Rx	8	81.914	1.633		
SNB	Pre Rx	8	77.381	1.951	-1.043	0.008*
	Post Rx	8	78.424	2.401		
ANB	Pre Rx	8	4.843	1.480	1.356	<0.001*
	Post Rx	8	3.486	1.612		
MPA	Pre Rx	8	23.704	4.133	-0.856	0.11
	Post Rx	8	24.560	4.328		
Gonial Angle	Pre Rx	8	121.671	3.344	-1.320	0.13
	Post Rx	8	122.991	5.302		

Skeletal Linear Parameters

Table 14 shows the mean values of Linear Skeletal Parameters between Pre Rx & Post Rx Period in Group 2. Point A perp to N value changed from 0.17 ± 4.77 to -0.53 ± 5.00 mm, with no statistical significance (P:0.67). The ANS perp. N value increased from 4.74 ± 2.72 mm to 3.82 ± 2.71 mm, which was not statistically significant (P:0.07). On comparing the difference between pre and post Rx value of ANS-PNS, the value changed from 50.10 ± 3.44 to 50.39 ± 3.85 mm respectively, with no statistical significance (P:0.09). The N perp. Pog value increased from -4.06 ± 6.95 mm to -3.38 ± 5.22 mm, which was not statistically significant (P:0.40). The change in the Point B perp. N from -4.75 ± 5.91 mm to -5.12 ± 5.14 mm, was found to have not statistically significance (P:0.96). The effective condylar length Co-Gn increased from 101.11 ± 2.56 mm to 102.57 ± 2.57 mm, which was statistically significant (P value: 0.01). On comparing the difference between pre and post Rx value of Go-Gn, the value changed from 67.14 ± 3.64 to 67.06 ± 3.50 mm respectively, with no statistical significance (P:0.89). The results suggest that there was significant increase in the effective mandibular length post AdvanSync appliance therapy (P-value: 0.01).

**Table 14:** Comparison of mean values of linear skeletal parameters between pre Rx & post Rx period in Group 2 using student paired t-test A PERP N: Point A perpendicular to Nasion, ANS PERP N: Anterior Nasal Spine perpendicular to Nasion, ANS-PNS: Anterior Nasal Spine-Posterior Nasal Spine, POG-PERP-N: Pogonion - perpendicular-Nasion, B-perp: B Perpendicular, CO-GN: Condylion - Gnathion, GO-GN: Gonion - Gnathion

Parameters	Time	N	Mean	SD	Mean Diff	P-value
A PERP N	Pre Rx	8	0.171	4.770	0.706	0.67
	Post Rx	8	-0.535	5.005		
ANS PERP N	Pre Rx	8	4.740	2.720	0.919	0.07
	Post Rx	8	3.821	2.710		
ANS-PNS	Pre Rx	8	50.100	3.863	-0.291	0.09
	Post Rx	8	50.391	3.859		
POG PERP N	Pre Rx	8	-4.066	6.951	-0.682	0.40
	Post Rx	8	-3.384	5.225		
B PERP	Pre Rx	8	-4.755	5.917	0.370	0.96
	Post Rx	8	-5.125	5.143		
CO-GN	Pre Rx	8	101.118	2.562	-1.459	0.01*
	Post Rx	8	102.576	2.579		
GO-GN	Pre Rx	8	67.144	3.644	0.080	0.89
	Post Rx	8	67.064	3.507		

Dental Angular Parameters

U1-SN angle reduced from 113.43 ± 7.90 to 108.64 ± 8.30 degrees which was statistically significance (P:<0.001). L1-SN angle which reduced from 101.71 ± 8.23 to 108.32 ± 8.53 degrees, with significant statistical difference (P:001). The L1-MP value increased from 99.76 ± 4.12 to 104.05 ± 4.39 degrees, which was statistically significant (P: <0.001). In the Advansync group, the upper incisors retroclined and the lower incisors proclined significantly.

Dental Linear Parameters

U1-NF value increased from 23.80 ± 2.08 to 24.91 ± 2.45 mm, which was found to be statistically significant (P:0.01). U6-NF value increased from 20.41 ± 2.96 to 20.70 ± 2.28 mm, with no statistical significance (P:0.61). The L1-MP value increased from 36.67 ± 2.74 to 37.77 ± 2.21 mm and this difference was not statistically significant. L6-MP value changed from 28.37 ± 4.63 to 27.82 ± 2.50 mm, with no statistical significance (P:0.62). The value for Molar correction changed from 1.80 ± 0.67 to -2.47 ± 1.12 mm, which was highly significant (P: <0.001). The Overjet reduced from 7.32 ± 2.70 to 3.28 ± 1.43 mm, which was found to be highly significant (P:0.01). The Overbite changed from 4.16 ± 0.89 to 2.35 ± 0.44 mm which was not statistically significant. The dentoalveolar changes that were found in AdvanSync group were extrusion of upper incisors, improvement in molar relationship, and reduction in overjet.

**Table 15:** Comparison of mean values of angular and linear dental parameters between pre Rx & post Rx period in Group 2 using student paired t-test U1-SN: upper incisor to sella-nasion, L1-SN: lower incisor to sella-nasion, L1-MP: lower incisor- mandibular plane, U1-NF: upper incisor- nasal floor, U6-NF: upper molar- nasal floor, L6-MP: lower molar- mandibular plane

Parameters	Time	N	Mean	SD	Mean Diff	P-value
U1-SN	Pre Rx	8	113.438	7.909	4.795	<0.001*
	Post Rx	8	108.643	8.304		
L1-SN	Pre Rx	8	51.039	7.109	3.760	0.01*
	Post Rx	8	47.279	8.823		
L1-MP	Pre Rx	8	101.711	8.236	-6.611	0.001*
	Post Rx	8	108.323	8.530		
U1-NF	Pre Rx	8	23.800	2.088	-1.119	0.01*
	Post Rx	8	24.919	2.459		
U6-NF	Pre Rx	8	20.414	2.967	-0.289	0.61
	Post Rx	8	20.703	2.228		
L1-MP	Pre Rx	8	36.679	2.745	-1.096	0.34
	Post Rx	8	37.775	2.216		
L6-MP	Pre Rx	8	28.379	4.634	0.557	0.62
	Post Rx	8	27.821	2.501		
Molar correction	Pre Rx	8	1.805	0.672	4.279	<0.001*
	Post Rx	8	-2.474	1.122		
Overjet	Pre Rx	8	7.328	2.709	4.045	0.01*
	Post Rx	8	3.283	1.433		
Overbite	Pre Rx	8	2.939	2.852	-0.040	0.97
	Post Rx	8	2.979	1.097		

Soft Tissue Angular Measurements

The facial convexity increased from 157.23 ± 5.39 to 159.63 ± 5.48 degrees, which was statistically significant (P:0.008). Similarly, Total facial convexity increased from 128.99 ± 5.36 to 131.54 ± 5.29 degrees, which was statistically significant (P:0.03). The Nasolabial angle changed from 100.64 ± 9.24 to 99.86 ± 11.55 degrees, with no statistical significance. The mentolabial sulcus changed from 103.49 ± 23.71 to 104.09 ± 20.31 degrees, with no statistical significance. Significant changes were found in the Facial convexity angle and Total facial convexity angle which resulted in improvement in the soft tissue facial profile.

The results suggest that AdvanSync appliance brought about significant change in the facial convexity angle and Total facial convexity, whereas no change was found in the Nasolabial and Mentolabial sulcus. Table 16 shows comparison of mean values of Angular Soft tissue Parameters between Pre Rx & Post Rx Period in Group 2.

**Table 16:** Comparison of mean values of angular facial soft tissue parameters between pre Rx & post Rx period in Group 2 using student paired t-test

Parameters	Time	N	Mean	SD	Mean Diff	P-value
Facial convexity	Pre Rx	8	157.231	5.395	-2.403	0.008*
	Post Rx	8	159.634	5.480		
Total facial convexity	Pre Rx	8	128.995	5.368	-2.553	0.03*
	Post Rx	8	131.548	5.298		
Nasolabial angle	Pre Rx	8	100.643	9.247	0.779	0.65
	Post Rx	8	99.864	11.556		
Mentolabial sulcus	Pre Rx	8	103.499	23.719	-0.591	0.90
	Post Rx	8	104.090	20.313		

## Discussion

Skeletal Class II malocclusion is one of the most common malocclusions in the general population. It occurs in about 25% to 30% of the general population. Moreover, mandibular retrognathism has been seen to be the most common causative factor for skeletal Class II malocclusion [7]. Functional appliances are well established as a means of correcting significant antero-posterior malocclusions [8]. To stimulate mandibular growth by the forward positioning of the mandible, various removable and fixed functional appliances are commonly used to alter the position of mandible [10]. The stimulation of mandibular growth, distal movement of the upper dentition, and mesial movement of the lower dentition contributes to the correction of Class II malocclusion with the use of fixed functional appliances [10]. The various treatment options for Class II correction apart from myofunctional/FFAs include orthopedic appliances like headgears, camouflage line of treatment by extractions of premolars, distalization of the maxillary arch or the surgical correction of the underlying skeletal discrepancy when growth has completed. For Class II growing patients in whom the mandible is retrognathic, the ideal means of correction is to target the source and try to alter the amount or direction of growth in that jaw. A wide range of functional appliances aimed to stimulate mandibular growth by forward posturing of the mandible are available to correct class II skeletal and occlusal disharmony [11]. FFAs are the most commonly used appliance to correct skeletal discrepancy in a growing stage, but still its enhancement effect on mandibular growth has been questionable [7].

The Forsus Fatigue Resistant Device (FRD) is a semirigid telescoping system incorporating a superelastic nickel titanium coil spring that can be assembled chair-side and that can be used in conjunction with complete fixed orthodontic appliances. Forsus appliance has proven to induce significant skeletal and dental changes, which remained relatively stable during the observation period in post-pubertal patients [12]. The AdvanSync appliance has the advantage of being used simultaneously with fixed mechanotherapy from the initial stage of treatment and claims to reduce the overall treatment duration. It also claims to eliminate the need for patient compliance and provides maximum comfort and range of movement. AdvanSync appliance is shown to have a significant maxillary sagittal restriction effect along with significant dentoalveolar effects [13].

Berco et al. found that CBCT allows for clinically accurate and reliable three-dimensional linear measurements of the craniofacial complex. Moreover, skull orientation during CBCT scanning does not affect the accuracy or the reliability of these measurements [14]. 

The purpose of our study was to assess and compare whether AdvanSync gives better skeletal, dental and soft tissue outcomes than Forsus fixed-resistant device for the correction of skeletal class II malocclusion in post-pubertal patients using 3-D CBCT.

The baseline parameters between the groups were statistically not significant, which shows that the type of cases and the severity of malocclusion of the subjects included in the study groups were similar at the pre-treatment stage (Tables 1-4). We can conclude that, in the current study, the bias was minimized.

When comparison was made between the two study groups, the skeletal, dental and soft tissue parameters did not show any statistical significance. Forsus and AdvanSync appliance showed similar post treatment results, which were mainly dentoalveolar in nature with mild changes in the effective mandibular length and soft tissues (Tables 5-8).

The appliance can be effectively used to correct skeletal class II malocclusion in post pubertal patients. These findings are consistent with previous studies on treatment effects of fixed functional appliances [15,16].

Zymperdikas et al. in a systematic review, concluded that FFAs seem to be effective in improving Class II malocclusion in the short term, although their effects seem to be mainly dentoalveolar and soft tissue changes rather than skeletal [15]. Similar results were obtained in current study wherein minimal skeletal sagittal effects were observed with improvement in SNB and ANB angles. However, there was no significant change in the SNA angle in the current study which contradicts the finding.

Shahi et al. found that the AdvanSync appliance resulted in inducing more changes in SNB and effective mandibular length as compared to Twin Block. This effective result with AdvanSync appliance may be attributed due to the effective full-time wear obtained whereas patient compliance may have been compromised with the Twin Block appliance. There was also reduction in SNA angle which was not significant [17]. Similar findings were found in the present study. The decrease in SNA could be explained by the distal reciprocal force exerted on the maxilla by the appliance. Both the appliances showed similar dentoalveolar changes. These findings are consistent with the current study, but results must be carefully interpreted since the study sample included patients in pubertal and post-pubertal growth phase.

Another literature review conducted by Linjawi and Abbassy revealed that the Forsus appliance showed positive effects on the maxillary incisors and first molars as well as overjet and overbite whereas multiple negative effects were reported on the occlusal plane and lower incisors which included protrusion, proclination, and intrusion of the lower incisors that need to be considered when using such appliance in treating Class II malocclusion [5]. In contrary, we found no significant effect on the maxillary incisors and maxillary molars in the vertical plane in the Forsus appliance group. Whereas the other dental parameters including overjet, overbite and sagittal molar relationship improved. And we also observed significant intrusion and worsening of the lower incisor inclination.

Jayachandran et al. concluded that AdvanSync and intermaxillary elastics were effective in class II corrections. AdvanSync produced its effects through maxillary skeletal growth restriction and mandibular dentoalveolar changes whereas class II elastics worked primarily through dentoalveolar changes [13]. The findings of the current study contradict the results of the above-mentioned study as there was no significant maxillary growth restriction effect in the AdvanSync group. Whereas the effects on the mandible and the dentoalveolar effects were found to be similar. These variations in observations could be contributed to the different methodologies used in the studies, variety of study designs, and that the patients’ age.

The AdvanSync appliance resulted in significant extrusion and retroclination of upper incisor, proclination of lower incisors, improvement in molar relation and overjet. These findings are in agreement with earlier studies that evaluated the dentoalveolar effects of functional appliances [13,15-17]. There was an improvement in the facial soft tissue convexity in both Forsus and AdvanSync appliance group in the current study.

Limitations

The treatment changes at the end of the comprehensive treatment were not assessed in the current study. The long-term stability of the fixed functional appliances was not evaluated. Some adverse effects with the AdvanSync appliance were observed immediately after removal of the appliance which included molar rotation and intrusion. These aspects can be further evaluated.

## Conclusions

There were no differences between the skeletal, dental, and soft tissue treatment effects between the Forsus and AdvanSync fixed functional appliances when used in post-pubertal patients. Both the appliances showed significant changes in the SNB, ANB angle, and effective mandibular length. The dentoalveolar effects of the Forsus appliance were significantly lower incisor proclination and intrusion, improvement in molar relation, and reduction in overjet and overbite.

The dentoalveolar effects seen with the AdvanSync appliance were retroclination and extrusion of the upper incisors, proclination of lower incisors, improvement in the molar relation, and reduction in overjet. The total tissue facial convexity increased in the Forsus group. In the AdvanSync group, both the facial convexity angle and total facial convexity angle increased resulting in improvement in soft tissue facial profile. Both the appliances exert their effect by a slight change in the mandibular skeletal parameters and majorly dentoalveolar effects.
